# Evaluation of the Role of *SNCA* Variants in Survival without Neurological Disease

**DOI:** 10.1371/journal.pone.0042877

**Published:** 2012-08-13

**Authors:** Michael G. Heckman, Alexandra I. Soto-Ortolaza, Nancy N. Diehl, Minerva M. Carrasquillo, Ryan J. Uitti, Zbigniew K. Wszolek, Neill R. Graff-Radford, Owen A. Ross

**Affiliations:** 1 Biostatistics Unit, Mayo Clinic, Jacksonville, Florida, United States of America; 2 Department of Neuroscience, Mayo Clinic, Jacksonville, Florida, United States of America; 3 Department of Neurology, Mayo Clinic, Jacksonville, Florida, United States of America; Oslo University Hospital, Norway

## Abstract

**Background:**

A variety of definitions of successful aging have been proposed, many of which relate to longevity, freedom from disease and disability, or preservation of high physical and cognitive function. Many behavioral, biomedical, and psychological factors have been linked with these various measures of successful aging, however genetic predictors are less understood. Parkinson's disease (PD) is an age-related neurodegenerative disorder, and variants in the α-synuclein gene (*SNCA*) affect susceptibility to PD. This exploratory study examined whether *SNCA* variants may also promote successful aging as defined by survival without neurological disease.

**Methods:**

We utilized 769 controls without neurological disease (Mean age: 79 years, Range: 33–99 years) and examined the frequency of 20 different *SNCA* variants across age groups using logistic regression models. We also included 426 PD cases to assess the effect of these variants on PD risk.

**Results:**

There was a significant decline in the proportion of carriers of the minor allele of rs10014396 as age increased (P = 0.021), from 30% in controls younger than 60 to 14% in controls 90 years of age or older. Findings were similar for rs3775439, where the proportion of carriers of the minor allele declined from 32% in controls less than 60 years old to 19% in those 90 or older (P = 0.025). A number of *SNCA* variants, not including rs10014396 or rs3775439, were significantly associated with susceptibility to PD.

**Conclusions:**

In addition to its documented roles in PD and α-synucleinopathies, our results suggest that *SNCA* has a role in survival free of neurological disease. Acknowledging that our findings would not have withstood correction for multiple testing, validation in an independent series of aged neurologically normal controls is needed.

## Introduction

Aging can be defined as a progressive, generalized impairment of function resulting in a loss of adaptive response to stress and an increasing risk of age-associated disease [1], [2]. Within this definition lies the paradoxical idea that individuals can age in a healthy manner, so-called successful aging, which is a topic that has received increased attention as a result of the dramatic increase in human life expectancy that has been witnessed during the past century. While longevity is clearly a key part of aging in a healthy manner, it is equally clear that it is not the only component, and that other factors require consideration [3]. The concept of successful aging was introduced by Rowe and Kahn, distinguishing “usual aging” from “successful aging”, and referring to successful aging as freedom from disease and disability, preservation of high physical and cognitive function, and engagement in social and productive activities [4], [5]. A number of other definitions of successful aging have been put forth, many of which, in addition to longevity and disease, emphasize disability, physical, or cognitive functioning [6], [7].

Many different attempts have been made to identify characteristics associated with the various measures of successful aging that have been proposed. The majority of these reports have investigated demographic, behavioral, biomedical, or psychological factors, and have identified predictors such as absence of arthritis, better activities of daily living, and not smoking, among many others [6], [7]. However, fewer studies have examined genetic predictors. The identification of genetic factors related to specific aspects of successful aging has several potential benefits, for instance, shedding light on the underlying biological systems and processes involved in aging successfully, and possibly allowing for the identification of people who possess harmful variants of those genes, for whom intervention could potentially increase the likelihood of successful aging.

Due to their links to specific aspects of successful aging such as disease, physical functioning, and cognitive functioning, the study of age-related disorders offers promise in further understanding the mechanisms involved in the aging process. Parkinson's disease (PD) is one of the most prevalent age-related neurodegenerative disorders, with approximately 1.5% of the population older than sixty-five years being affected [8], [9], and at the center of genetic PD research is the α-synuclein gene (*SNCA*). Point mutations in *SNCA* were the first mutations identified in familial PD [10]. Subsequent studies showed α-synuclein to be the major protein component of the pathologic inclusion observed in PD, the Lewy body 11,12. Identification of genomic multiplication of the *SNCA* gene in familial PD demonstrated that over-expression could cause disease in a dose-dependent manner [13], [14]. This observation fitted with the theory that common variation at the *SNCA* locus increased risk of sporadic PD via a pathomechanism of limited over-expression [15]–[19].

We speculated that variation in age-related disease genes such as *SNCA* may not only affect morbidity, but also influence particular aspects of successful aging. More specifically, in addition to its role in PD, we hypothesized that variation in *SNCA* may be associated with neurological disease in general and longevity. Therefore, the primary aim of this exploratory study was to evaluate the association between common *SNCA* single nucleotide polymorphisms (SNPs) and survival without neurological disease.

## Methods and Subjects

### Ethics statement

The Mayo Clinic institutional review board approved all work. All participants provided written informed consent.

### Subjects

In evaluating the role of common *SNCA* variants in successful aging as measured by survival without neurological disease, we utilized a series of 769 controls without neurological disease seen at Mayo Clinic Florida. Unrelated controls were deemed to be free of neurological disease by a neurologist, and did not have a family history of neurological disease. Mean age in the controls was 79±11 SD years (Range: 33–99 years), and 385 (50%) were male. All controls were white and of European ancestry. Controls were mostly unrelated spouses/caregivers of neurological patients, and a subset of these controls are included in our aging sibpair study [20]. Utilizing this group of controls, we evaluated the association of *SNCA* SNPs with age, where since the probability of living longer without neurological disease increases as age without neurological disease increases, age in these controls is a measure of survival without neurological disease.

We supplemented the healthy control series with 426 PD cases also seen at Mayo Clinic Florida, in order to examine associations of *SNCA* SNPs with PD [21]. Mean age of PD cases was 72±11 SD years (Range: 30–92 years), mean age at PD onset was 62±12 SD years (Range: 26–85 years), and 233 (55%) were male. All PD cases were white and of European ancestry, and were examined and observed longitudinally by a neurologist and diagnosed with PD according to published criteria [22]. There are no known related samples within or between the diagnosis groups. All subjects self-identified ethnicity.

### Genetic analysis

DNA was extracted from whole blood samples using standard protocols. Conserved regions (conservation score >200) were identified across *SNCA* (coding regions ±10 Kb) using the phastConst software embedded in UCSC Genome Browser (http://genome.ucsc.edu), based on the NCBI March 2006 assembly [23]. The conserved SNP approach was used to highlight potentially functional variation and takes into account the high levels of linkage disequilibrium at the *SNCA* locus. We identified 20 SNPs with a minor allele frequency >1% which were selected for analysis. The *SNCA* variants were genotyped on a Sequenom MassArray iPLEX platform (San Diego, CA; primer sequences are available on request) and analyzed with Typer 4.0 software. The rate of genotype calls was ≥95% for all SNPs.

### Statistical analysis

Deviations of SNP genotypes from Hardy Weinberg equilibrium (HWE) in controls were assessed using chi-square tests, while linkage disequilibrium (LD) between SNPs in controls was assessed using *r^2^* values. In evaluation of the primary study aim, we used logistic regression models adjusted for gender to examine whether the proportion of carriers of the minor allele for each SNP varied across age in controls, where age was considered as a continuous variable. After examination of genotype frequencies in the overall group of controls, we chose to consider each SNP under a dominant model (i.e. presence or absence of the minor allele) owing to the relatively small number of homozygotes of the minor allele for many of the *SNCA* SNPs. We also divided age into categories, and estimated the proportion of control carriers of the minor allele in each age category. When utilizing the overall case-control series, single SNP associations with PD were evaluated using logistic regression models adjusted for age and gender under a dominant model. Odds ratios (ORs) and 95% confidence intervals (CIs) were estimated. Owing to the fact that the primary analysis comparing the frequency of *SNCA* variants across age is exploratory in nature in that this has not been evaluated before, no adjustment for multiple testing was made, and p-values of 0.05 or less were considered as statistically significant. All statistical analyses were performed using R statistical software (version 2.11.9; R Foundation for Statistical Computing, Vienna, Austria).

## Results

**Figure 1:**
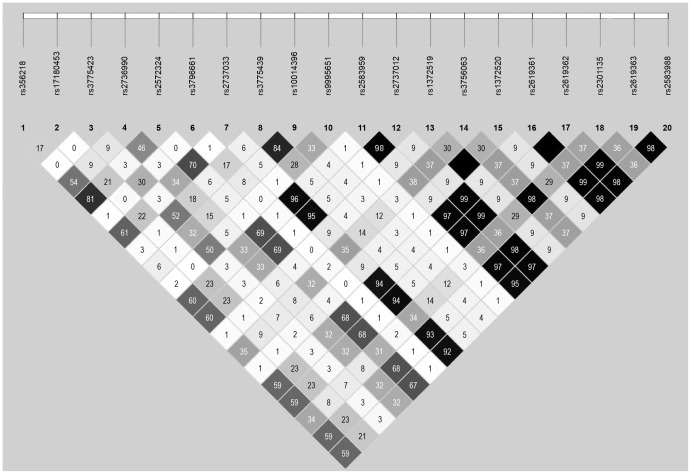
Linkage disequilibrium between SNPs in controls as measured by pairwise r^2^ values. Values are given as percentages out of a maximum of 100. Solid black boxes indicate an r^2^ value of 100.

There was no evidence of a departure from HWE for any of the SNPs (all P>0.05). LD between SNPs is summarized in Figure 1; there was moderate to strong LD between a number of *SNCA* SNPs, with many *r^2^* values of 0.30 or greater.

For each *SNCA* SNP, the proportion of carriers of the minor allele in controls is displayed in Table 1 for seven different age categories: <60 years, 60–69 years, 70–74 years, 75–79 years, 80–84 years, 85–89 years, and ≥90 years. These age categories were chosen with the goal of displaying trends in as detailed a manner as possible, while maximizing the number of controls in each category. As seen in Table 1, there was a significant decline in the proportion of carriers of the minor allele of rs10014396 as age increased (P = 0.021), where 30% of controls younger than 60 were carriers, compared to 14% of those 90 years of age and older. Results were similar for rs3775439, which was in strong LD with rs10014396 (*r^2^* = 0.84); the proportion of carriers of the minor allele decreased from 32% in controls younger than 60 to 19% in those 90 or older (P = 0.025). For all remaining *SNCA* SNPs, the proportion of carriers of the minor allele did not noticeably increase or decrease as age increased in controls (all P≥0.19).

**Table 1:** Proportion of carriers of the minor allele according to age in controls free of neurological disease.

			Proportion of carriers of the minor allele in controls							
			Age: <60 (N = 54)	Age: 60–69 (N = 91)	Age: 70–74 (N = 79)	Age: 75–79 (N = 89)	Age: 80–84 (N = 118)	Age: 85–89 (N = 261)	Age: ≥90 (N = 77)	
Chr4: (bp)*	SNP	Minor allele	No. (%)	No. (%)	No. (%)	No. (%)	No. (%)	No. (%)	No. (%)	P-value
90637010	rs356218	A	26 (48)	50 (55)	45 (57)	50 (56)	60 (51)	139 (53)	45 (58)	0.53
90653134	rs17180453	T	9 (17)	11 (12)	11 (14)	20 (22)	12 (10)	39 (15)	16 (21)	0.63
90657491	rs3775423	T	8 (15)	17 (19)	10 (13)	9 (10)	18 (15)	46 (18)	10 (13)	0.90
90678541	rs2736990	G	39 (72)	70 (77)	59 (75)	61 (69)	78 (66)	182 (70)	56 (73)	0.31
90678798	rs2572324	G	24 (44)	43 (49)	39 (49)	48 (54)	58 (49)	125 (48)	41 (53)	0.41
90687507	rs3796661	T	4 (7)	7 (8)	1 (1)	5 (6)	7 (6)	14 (5)	4 (5)	0.59
90707947	rs2737033	C	24 (44)	35 (38)	36 (46)	45 (51)	47 (40)	118 (45)	40 (52)	0.27
90709741	rs3775439	A	17 (32)	33 (36)	23 (29)	20 (22)	27 (23)	69 (26)	15 (19)	0.025
90712629	rs10014396	C	16 (30)	30 (33)	21 (27)	17 (19)	23 (19)	63 (24)	11 (14)	0.021
90716177	rs9995651	G	4 (7)	10 (11)	8 (10)	3 (3)	10 (8)	29 (11)	5 (6)	0.98
90721637	rs2583959	G	24 (44)	34 (37)	35 (43)	44 (49)	48 (41)	120 (46)	40 (52)	0.21
90745707	rs2737012	A	24 (44)	34 (37)	35 (44)	43 (48)	49 (42)	120 (46)	40 (52)	0.21
90757309	rs1372519	A	23 (43)	38 (42)	29 (37)	36 (40)	50 (42)	90 (34)	30 (39)	0.19
90757394	rs3756063	G	39 (72)	62 (68)	58 (73)	67 (75)	89 (75)	185 (71)	60 (78)	0.54
90757505	rs1372520	T	22 (42)	38 (42)	29 (37)	36 (40)	50 (42)	90 (34)	30 (39)	0.22
90757735	rs2619361	A	24 (44)	35 (38)	35 (43)	44 (49)	49 (42)	119 (46)	40 (52)	0.26
90757845	rs2619362	T	24 (44)	35 (38)	35 (43)	44 (49)	49 (42)	118 (45)	40 (52)	0.27
90758389	rs2301135	G	39 (74)	64 (71)	60 (78)	66 (75)	85 (72)	175 (67)	57 (74)	0.79
90759047	rs2619363	T	24 (44)	35 (38)	34 (43)	44 (49)	50 (42)	119 (46)	40 (52)	0.24
90760828	rs2583988	T	24 (44)	35 (38)	34 (43)	44 (49)	49 (42)	118 (45)	40 (52)	0.26

SNP = single nucleotide polymorphism. CI = confidence interval. *Chromosomal positions based on the February 2009 (GRCH37/hg19) genome assembly [*SNCA* is located at Chr4;90,645,251–90,759,447]. P-values result from logistic regression models adjusted for gender, where the outcome was presence of the minor allele of the given SNP, and the predictor variable was age as a continuous variable. Genotype call rates for all SNPs were >95%.

In Table 2 we present single SNP associations with PD in the entire series of 426 PD cases and 769 controls. Of particular interest, rs10014396 and rs3775439, both of which were associated with survival without neurological disease in controls, were not associated with PD (ORs 0.92 & 0.95, P≥0.58). Significant protective associations in relation to PD were observed for rs1372519 (OR: 0.76, P = 0.034) and rs1372520 (OR: 0.76, P = 0.039). For the remaining *SNCA* SNPs, a number of significant risk associations with PD were observed, including rs356218, rs2736990, rs2572324, rs2737033, rs2583959, rs2737012, rs2619361, rs2619362, rs2619363, and rs2583988 (ORs between 1.39 and 1.64, all P≤0.026), all of which were in moderate to strong LD (Figure 1).

**Table 2:** Single SNP associations with PD.

			Minor allele count and frequency			
Chr4: (bp)*	SNP	Minor allele	PD cases	Controls	OR (95% CI)	P-value
90637010	rs356218	A	335 (39%)	505 (33%)	1.49 (1.15, 1.92)	0.002
90653134	rs17180453	T	83 (10%)	124 (8%)	1.24 (0.89, 1.71)	0.20
90657491	rs3775423	T	84 (10%)	125 (8%)	1.31 (0.95, 1.82)	0.10
90678541	rs2736990	G	459 (54%)	724 (47%)	1.39 (1.04, 1.85)	0.026
90678798	rs2572324	G	303 (36%)	447 (29%)	1.42 (1.10, 1.82)	0.006
90687507	rs3796661	T	29 (3%)	44 (3%)	1.16 (0.69, 1.95)	0.57
90707947	rs2737033	C	295 (35%)	408 (27%)	1.64 (1.28, 2.10)	<0.001
90709741	rs3775439	A	121 (14%)	217 (14%)	0.95 (0.72, 1.25)	0.70
90712629	rs10014396	C	105 (12%)	188 (12%)	0.92 (0.69, 1.23)	0.58
90716177	rs9995651	G	47 (6%)	69 (4%)	1.30 (0.87, 1.96)	0.21
90721637	rs2583959	G	288 (34%)	401 (26%)	1.62 (1.27, 2.08)	<0.001
90745707	rs2737012	A	287 (34%)	399 (26%)	1.62 (1.27, 2.08)	<0.001
90757309	rs1372519	A	155 (18%)	339 (22%)	0.76 (0.58, 0.98)	0.034
90757394	rs3756063	G	442 (52%)	738 (48%)	1.30 (0.97, 1.74)	0.075
90757505	rs1372520	T	155 (18%)	338 (22%)	0.76 (0.59, 0.99)	0.039
90757735	rs2619361	A	287 (34%)	400 (26%)	1.61 (1.26, 2.06)	<0.001
90757845	rs2619362	T	94 (28%)	66 (23%)	1.61 (1.26, 2.07)	<0.001
90758389	rs2301135	G	436 (53%)	721 (49%)	1.24 (0.92, 1.68)	0.15
90759047	rs2619363	T	286 (34%)	400 (26%)	1.59 (1.24, 2.04)	<0.001
90760828	rs2583988	T	283 (33%)	398 (26%)	1.56 (1.22, 2.01)	<0.001

PD = Parkinson's disease. SNP = single nucleotide polymorphism. MA = minor allele. OR = odds ratio. CI = confidence interval. *Chromosomal positions based on the February 2009 (GRCH37/hg19) genome assembly [*SNCA* is located at Chr4;90,645,251–90,759,447]. ORs, 95% CIs, and p-values result from logistic regression models adjusted for age and gender. ORs correspond to presence vs. absence of the minor allele.

## Discussion

Lifespan in humans has been shown to display markable heritability, with estimates ranging between 20% and 30%, confirming that longevity is to some extent determined by genetic mechanisms [24], [25]. Identification of genetic variation influencing population mortality and specific aspects of successful aging may help characterize the overarching pathways and physiological processes that determine risk for common age-related diseases. Conversely, the study of age-related disease may provide greater insight into the molecular mechanisms that contribute to the aging process. In this exploratory study involving more than 750 controls free of neurological disease, our findings indicate that in addition to its known role in PD, *SNCA* may play a part in determining whether or not a given individual ages successfully as measured by the specific outcome of survival without neurological disease. More specifically, for two *SNCA* SNPs, rs10014396 and rs3775439, the minor allele was less common in older controls. The lower proportion of carriers of the minor allele for these two SNPs in the older controls suggests that unobserved individuals who are carriers of these alleles experience death or neurological disease at an earlier age and are therefore removed from the pool of controls free of neurological disease at later ages, indicating that the presence of these alleles increases the risk of neurological disease or death. The two age-associated *SNCA* SNPs were not among those that were associated with PD when supplementing the controls with the PD cases in disease-association analysis; this may be due to the different outcome measure used which relates not only to PD but to death and neurological disease in general.

Similar to the results of our study, other studies have demonstrated associations between genes involved in neurodegenerative disorders and successful aging outcomes. As described by Glatt et al. in their review of 29 different studies evaluating at least one other characteristic of healthy aging in addition to longevity [26], *APOE*, the known risk factor for Alzheimer's disease, has been implicated in successful aging in several studies, however this association has not been consistently identified, and further study is therefore needed. Walter et al., in their genome-wide association study [27], identified evidence of association with survival free of major disease for *GRIN2B*, which has been implicated in Parkinson's disease [28] and Alzheimer's disease [29]. These findings, taken together with those from our study, suggest that the role of age-related neurodegenerative disease genes should be further studied in relation to healthy aging in general.

Although not of primary interest in this study, it is worth highlighting the significant associations of *SNCA* SNPs with PD that were observed in this study and previous studies [15]–[19], [21]. Protective effects were observed for rs13752519 and rs1372520, which were in strong LD, while risk effects were also noted for a number of other SNPs that were also in relatively high LD. In addition to their known role in PD, *SNCA* variants have also been implicated in both diffuse Lewy body disease [30] and multiple system atrophy [31], [32], [33]. The associations between *SNCA* variants and survival without neurological disease that were observed in this study provide additional support for all of these findings and also further underscore the pleiotrophic nature of *SNCA*.

Several limitations are important to take into account in the interpretation of our results. There are other factors that are likely associated with survival free of neurological disease for which we did not collect information, and as such we cannot rule out the possibility that the associations of *SNCA* with survival free of neurological disease that we identified may be due in part to confounding factors. Also, owing to the exploratory nature of the study, we made no adjustment for multiple testing. The statistically significant decreasing frequencies of the minor allele for rs10014396 and rs3775439 as age increased in controls would not have remained significant after such an adjustment (P≤0.0058 considered significant after permutation adjustment for multiple testing), and therefore it is important to highlight that our findings require validation in an independent series of healthy individuals. Related to this, our sample size of more than 700 controls free of neurological disease is relatively small, and therefore the possibility of Type II error (i.e. a false negative association), particularly after adjustment for multiple testing, is important to consider. Larger series' of healthy controls, in particular healthy nonagenarians and centenarians, are needed in future evaluation of *SNCA*'s role in the aging process. Finally, an important caveat of genetic aging studies is the possibility of population stratification given migration patterns and generational differences in lifestyle.

Acknowledging these limitations, our results indicate that in addition to its known role in PD and α-synucleinopathies, *SNCA* may influence the likelihood of survival without neurological disease. Our findings, though requiring validation in an independent series, suggest that further study of the role of *SNCA* variation in aging and cognitive function is warranted.
